# A novel 19-gauge integrated electrocautery-puncture needle for endoscopic ultrasound-guided biliary drainage

**DOI:** 10.1055/a-2731-3448

**Published:** 2025-11-10

**Authors:** Tingting Yu, Suning Hou, Yongzhan Zhao, Linghan Xue, Xiaohan Xu, Senlin Hou, Lichao Zhang

**Affiliations:** 1 71213 The Second Hospital of Hebei Medical University, Shijiazhuang, China

Endoscopic ultrasound-guided biliary drainage (EUS-BD) has been established as a primary drainage modality for malignant biliary obstruction, with expanded clinical indications 1 2 . Following successful puncture of the target bile duct with a 19-gauge needle and guidewire placement, replacement of the needle with a cystotome for dilation of the fistula tract may be required 3 4 , which increases procedural complexity. Here, we designed a novel 19-gauge integrated electrocautery-puncture needle with puncture and dilation functions to address this challenge ( Fig. 1 ).

**Fig. 1:**
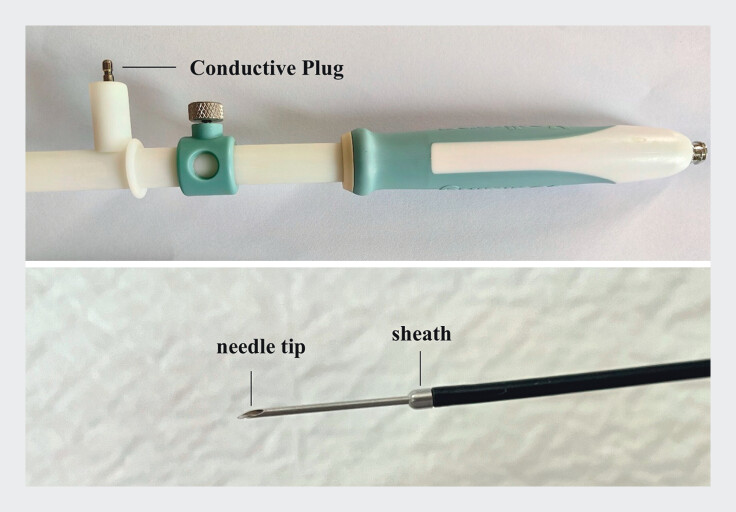
The display of a novel 19-gauge integrated electrocautery-puncture needle with an 8.5 French sheath.

A 68-year-old woman was admitted to our hospital with jaundice and was diagnosed with a distal malignant biliary stricture. She had undergone a total gastrectomy with Roux-en-Y reconstruction for gastric cancer 2 years ago. Due to her cachexia, ascites, and the high risk of perforation associated with prolonged ERCP procedures, we opted for EUS-BD as the preferred intervention. The dilated bile duct in segment II of the liver was selected as the target bile duct, with a diameter of approximately 6.5 mm and a puncture depth of 24 mm ( Fig. 2 **a** ). The novel 19-gauge integrated electrocautery-puncture needle was used to puncture the selected bile duct ( Fig. 2 **b** ). Thereafter, a 0.025-inch guidewire (Jagwire, Boston Scientific, USA) was advanced into the left intrahepatic duct, and then to the common bile duct, and finally into the intestinal cavity. Following needle withdrawal into the sheath, the sheath is advanced along the guidewire to dilate the fistula ( Fig. 2 **c** , Fig. 3 **a, b** , Video 1 ). Finally, a fully covered self-expandable metal stent (FCSEMS) was advanced over the guidewire to traverse the stricture and then papilla (EUS-guided antegrade stenting), and a 7-Fr and 10-cm-long double-pigtail plastic stent was inserted into the left hepatic duct (EUS-guided hepaticogastrostomy) ( Fig. 3 **c** ).

**Fig. 2:**
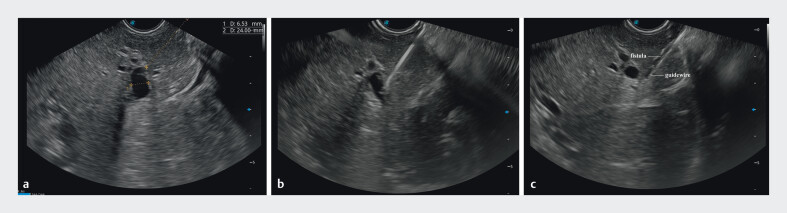
Endoscopic ultrasound images. **a** The left dilated intrahepatic bile duct (segment 2) and puncture pathway. **b** The target bile duct was punctured using a novel 19-gauge needle. **c** The fistula tract dilated by the sheath.

**Fig. 3:**
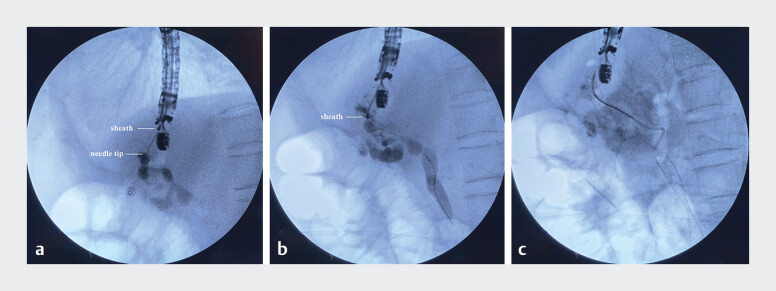
Fluoroscopic images. **a** The access of the needle tip through the digestive wall into the intrahepatic bile duct. **b** The activated sheath was advanced along the guidewire into the bile duct. **c** The released fully covered self-expandable metal stent and plastic stent.

A novel 19-gauge integrated electrocautery-puncture needle applied in EUS-BD.Video 1

The novel 19-gauge integrated electrocautery-puncture needle eliminates the need for exchanging the cystotome during EUS-BD procedures, thereby mitigating the risk of guidewire dislodgement and reducing procedural time.

Endoscopy_UCTN_Code_TTT_1AS_2AH

## Notes

Endoscopy E-Videos https://eref.thieme.de/e-videos
*E-Videos* is an open access online section of the journal *Endoscopy* , reporting on interesting cases and new techniques in gastroenterological endoscopy. All papers include a high-quality video and are published with a Creative Commons CC-BY license. Endoscopy E-Videos qualify for HINARI discounts and waivers and eligibility is automatically checked during the submission process. We grant 100% waivers to articles whose corresponding authors are based in Group A countries and 50% waivers to those who are based in Group B countries as classified by Research4Life (see: https://www.research4life.org/access/eligibility/ ). This section has its own submission website at https://mc.manuscriptcentral.com/e-videos .
